# Tumor Infiltrating Lymphocytes and Macrophages Improve Survival in Microsatellite Unstable Colorectal Cancer

**DOI:** 10.1038/s41598-019-49878-4

**Published:** 2019-09-17

**Authors:** Sumana Narayanan, Tsutomu Kawaguchi, Xuan Peng, Qianya Qi, Song Liu, Li Yan, Kazuaki Takabe

**Affiliations:** 1 Department of Surgical Oncology, Roswell Park Comprehensive Cancer Center, Buffalo, NY USA; 2 Department of Biostatistics & Bioinformatics, Roswell Park Comprehensive Cancer Center, Buffalo, NY USA; 3 Department of Surgery, Jacobs School of Medicine and Biomedical Sciences, University at Buffalo, The State University of New York, Buffalo, NY USA

**Keywords:** Cancer microenvironment, Surgical oncology

## Abstract

Due to the loss of DNA repair mechanisms in colorectal cancer (CRC) with microsatellite instability (MSI), somatic mutations accumulate within DNA; making them more prone to attack by tumor infiltrating lymphocytes (TIL) and macrophages. We hypothesize that MSI-High (MSI-H) patients have favorable survival due to increased tumor immunogenicity. The Cancer Genome Atlas (TCGA) was used to evaluate gene expression from 283 patients with CRC, comparing MSI-H and microsatellite stable (MSS) patients. CIBERSORT algorithm estimated the fraction of immune cell types. We found that low expression of DNA repair genes (*MLH1, MLH3, PMS1, PMS2, ATR, PRKDC, ATM, BRCA2*) associated with MSI-H. MSI-H was directly associated with Helper T-cells (*p* = *0.034*) and M1 macrophages (*p* < *0.0001*). MSI-H tumors associated with diminished intra-tumoral heterogeneity as well as higher expression of checkpoint molecules PD-1, PD-L1, CTLA4, LAG3 and TIM3 (*p* < *0.0001*). Improved OS was seen in patients with low ATM, PMS2 and MLH3. In the TCGA CRC cohort, decreased expression of DNA repair genes associated with MSI-H. MSI-H patients had improved survival, likely due to higher TIL and M1 macrophage infiltration as well as lower intra-tumoral heterogeneity. MSI-H also associates with expression of immune checkpoint molecules with potential for development of therapeutic targets.

## Introduction

Colorectal cancer (CRC) is the third most commonly occurring cancer and the fourth most common cause of cancer death worldwide^1–4^. A common hallmark in the development of CRC as in other cancers is defective DNA repair. Previous studies have linked up-regulation of DNA repair genes to poor prognostic factors such as resistance to chemotherapy and radiation as well as metastatic ability in tumors^5^. The DNA mismatch repair (MMR) pathway is important for correcting incorrect nucleotide insertions, deletions and substitutions^5^.

Microsatellite instability (MSI) occurs sporadically via inactivation of MMR genes by hypermethylation of their promoter regions resulting in impaired DNA repair function and accumulation of abnormal genes or via germ-line mutations in MMR genes (Lynch syndrome)^6,7^. Approximately 15% of CRC’s are deficient in MMR genes which include *MLH1, PMS1, PMS2, MSH2, MSH6, MLH3 and MSH3*^8^. MSI-High (MSI-H) patients have been associated with improved survival compared to microsatellite stable (MSS) in patients with localized CRC^5,6^. In addition, immunotherapeutic agents have demonstrated improved disease control and progression free survival in patients with advanced or metastatic MSI-H CRC^9,10^.

This improved survival in MSI-H CRCs is hypothesized to be due to accumulation of somatic mutations within these tumors, resulting in subsequent immune cell infiltration into tumors^11,12^. Indeed, one of the clinicopathological criteria of MSI is high lymphocyte infiltration. This increased immunogenicity has also been predictive of diminished lymph node involvement, decreased incidence of distant metastases and increased chemo-responsiveness^13,14^.

In this study we aimed to identify whether low expression of DNA repair genes associated with MSI-H and with improved survival due to genomic instability, intra-tumoral immune cell infiltration and immunologic responsiveness. We also aimed to investigate whether improvement in survival correlated with diminished intra-tumoral heterogeneity.

## Methods

### Gene Expression Analysis

A cohort of 283 patients with colorectal cancer was obtained from The Cancer Genome Atlas (TCGA)^15–20^. Data obtained from these patients was deemed exempt from the Institutional Review Board at Roswell Park Comprehensive Cancer Center  since the patient data is de-identified and publicly available. RNA sequence gene expression quantification data for colon cancer was retrieved from the Genomics Data Commons (GDC) data portal. Gene expression levels were derived using normalization methods provided in the DESeq. 2 package and designated as low or high. The expression of DNA repair genes was then compared between MSI and Microsatellite stable (MSS) cohorts. These DNA repair genes included *ATM, PRKDC, BRCA1, BRCA2, ATR, LIG1, POLE, SLX4* as well as MMR genes *MSH6, MLH1, PMS1, PMS2 and MLH3* as these are the most frequently mutated DNA repair genes in colorectal cancer as well as all MMR genes as determined by Chae, Y.K., *et al*.^5^. In initial analysis, higher expression of *MSH2*, in contrast to all other MMR genes was associated with MSI-H and was thus excluded from further analyses.

### MSI Determination

Hause *et al*. examined 5,930 cancer exomes from 18 cancer types in TCGA data and designed a microsatellite instability classifier (MOSAIC) for MSI using instability calls. The classifier was then used to distinguish MSI-high (MSI-H) from MSI-stable (MSS) samples for TCGA data independently of cancer types^21^. Predicted MSI calls and intermediate results were obtained from Hause *et al*. for subsequent analysis; http://krishna.gs.washington.edu/content/members/hauser/mosaic/.

### Cytolytic Activity Score (CYT)

The immune cytolytic activity score (CYT) was defined as the geometric mean of Granzyme A (GZMA) and Perforin 1 (PRF1) expression values in Transcripts Per Million (TPM)^22–24^.

### Mutant-Allele Tumor Heterogeneity (MATH) score

Mutant-allele tumor heterogeneity (MATH) score, a measure of intra-tumor heterogeneity, was calculated through R/Bioconductor package “maftools”; efficient analysis, visualization and summarization of (MAF) files from large-scale cohort-based cancer studies (https://www.biorxiv.org/content/early/2016/05/11/052662)^25–27^. This technique was developed by Mroz *et al*. and uses whole exome sequencing of tumors with matched normal DNA to determine the fraction of sequenced DNA which shows the mutant allele or mutant-allele fraction (MAF)^26^.

### Determination of Tumor infiltrating Immune Cells

In order to differentiate the numerous cell types that compose the immune response we utilized the CIBERSORT deconvolution algorithm which uses a set of reference gene expression values as a representation of each cell type and identifies cell type proportions in data sets of colorectal tumor gene expression data obtained from TCGA (further described by Ali, H.R. *et al*.)^28^. Twenty-two cell types were investigated in this research using CIBERSORT its online calculator (https://cibersort.stanford.edu/).

### Gene Set Enrichment Analysis with TCGA

Gene Set Enrichment Analysis (GSEA) was performed using software provided by the Broad Institute (http://software.broadinstitute.org/gsea/index.jsp)^29^. All reported tests were conducted at a nominal significance level of 0.05. Statistical analyses were performed using R software (http://www.r-project.org/) and Bioconductor (http://bioconductor.org/).

### Statistical analysis

Patients were dichotomized to into low and high groups based on different expression levels of genes in interest. To determine the threshold of the dichotomization, running Cox proportional hazard statistics were applied. Differences in the overall survival (OS) or disease free survival (DFS) between the two groups were assessed at multiple candidate cutoffs within the range of gene expression level, and the optimal cut off point was chosen based on the statistical significance of the Cox proportional hazards model. To compare the survival curves of individual groups, the Kaplan-Meier method with log-rank test and Cox proportional hazard regression were used when appropriate. The reported results included hazard ratios (HR) and 95% confidence intervals (CI). Association between variables including MSI status, gene expression, cell composition and other clinical characteristics were accessed using Mann–Whitney U test.

All reported tests were conducted at a nominal significance level of 0.05. Statistical analyses were performed using R software (http://www.r-project.org/) and Bioconductor (http://bioconductor.org/).

## Results

### Patient demographics

Gene expression data was obtained from a cohort of 283 patients. Of these, 127 patients were female and 156 male with a mean age of 65. Within this cohort, 255 patients had data on MSI status; 204 patients were MSS and 51 patients were MSI-H. Staging data was available for 274 patients, with the majority being stage II (40.1%) and stage III (29.2%). 41.3% patients were node positive and 14% patients had known metastatic disease in the entire cohort. 54.4% of patients had tumors located within the right colon, 36.6% with left colon cancer and 9% had transverse colon adenocarcinoma. The MSI-H group had more Stage I (24% vs. 15%) and Stage II (56% vs. 35%) patients than the MSS group, which had more Stage III and IV patients (Table 1). More tumors within the right colon were MSI-H than MSS (75% vs. 48%).

**Table 1:** Clinical Variables of MSI-H compared to MSS patients.

Clinical Variables	MSI-H (%)	MSS (%)
Gender (F/M)	47/53	46/54
Age Mean (range)	69 (34–90)	65 (31–90)
Stage (I/II/III/IV)	24/56/16/4	15/35/33/17
T stage (T1/T2/T3/T4)	4/20/66/10	2/15/68/15
N stage (N0/N1/N2)	76/4/20	53/28/19
M stage (M0/M1/MX)	80/14/6	66/16/18
Primary location (Left/right/transverse)	10/75/15	43/48/9

### DNA repair gene expression was significantly lower in MSI-high tumors

**Figure 1:**
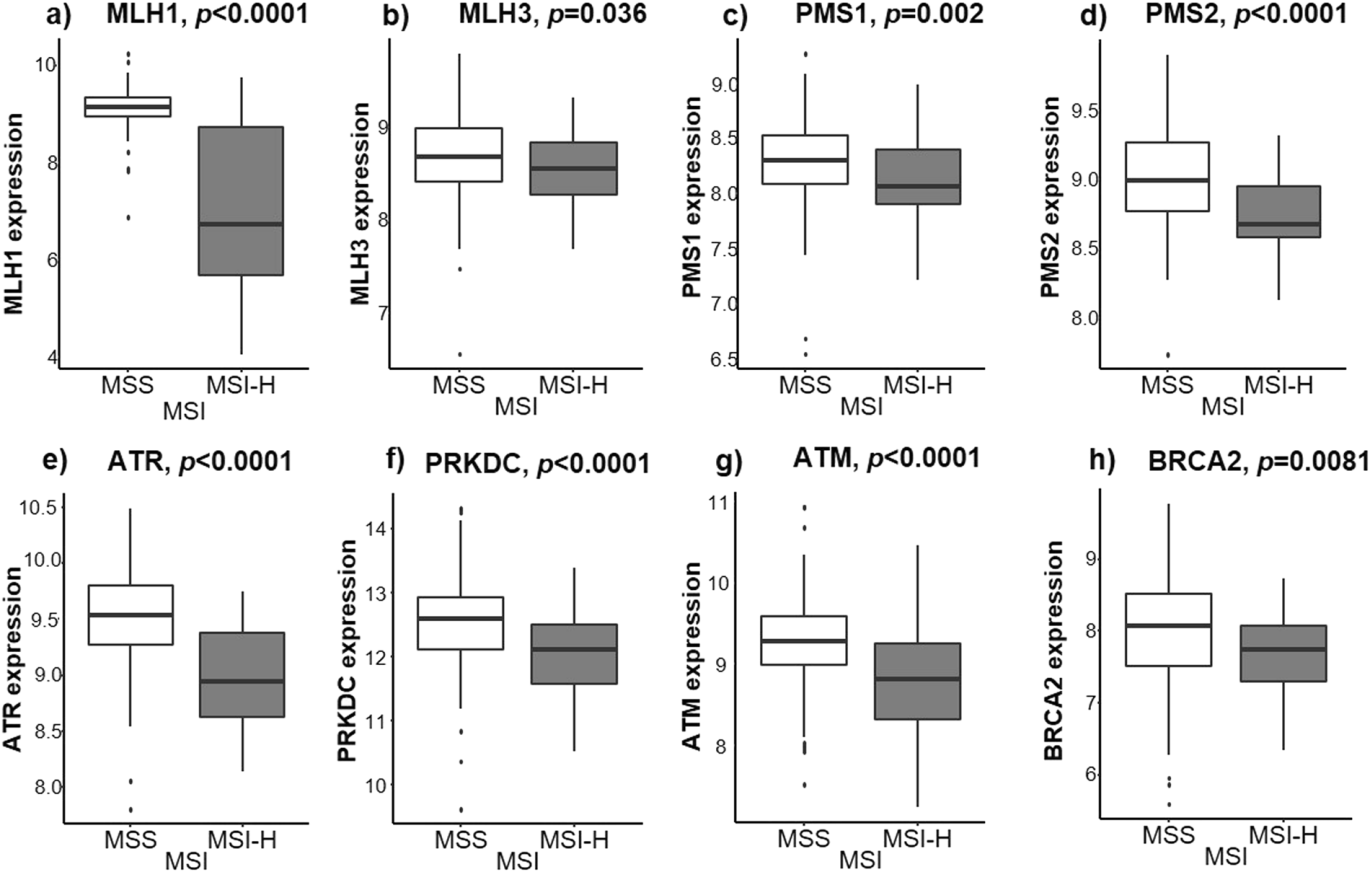
DNA repair gene expression in MSI-H compared to MSS patients: (**a**) MLH1, (**b**) MLH3, (**c**) PMS1, (**d**) PMS2, (**e**) ATR, (**f**) PRKDC, (**g**) ATM and (**h**) BRCA2.

We initially sought to determine whether MSI-high tumors have lower expression of DNA repair genes, which is the current dogma. As expected, expression (as noted by relative values) of eight DNA repair genes was significantly lower in MSI-high tumors. Some of these were the expected mismatch repair (MMR) genes- *MLH1* (6.74 vs. 9.15, *p* < *0.0001*), *MLH3* (8.55 vs. 8.68, *p* = *0.036*), *PMS1* (8.06 vs. 8.30, *p* = *0.002*) and *PMS2* (8.67 vs. 8.99, *p* < *0.0001*). Others included non-MMR double stranded break DNA repair genes such as *ATR* (8.94 vs. 9.53, *p* < *0.0001*), *PRKDC* (12.11 vs. 12.59, *p* < *0.0001*), *ATM* (8.81 vs. 9.28, *p* < *0.0001*) and *BRCA2* (7.74 vs. 8.07, *p* = *0.0081*), (Fig. 1a–h). Although they did achieve statistical significance, the difference in expression of *MLH3*, *PMS1* and *BRCA2* were not as dramatic as we expected from previous reports which may reflect the difference in methodology of using RNA sequencing data from TCGA.

### MSI-H tumors are associated with higher tumor mutation load and Cytolytic Activity Score (CYT) but lower Mutant-Allele Tumor Heterogeneity (MATH)

**Figure 2:**
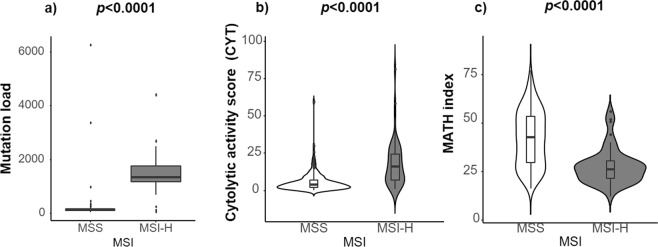
(**a**) Box plot comparing mutation load in MSI-H vs. MSS patients, (**b**) Jitter plot demonstrating Cytolytic activity score (CYT) in MSI-H vs. MSS and (**c**) Jitter plot demonstrating Mutant-Allele Tumor Heterogeneity (MATH) levels comparing MSI-H and MSS.

Next, we examined the mutation load, which we expected to be high in MSI-H tumors. As expected, MSI-H tumors had significantly higher mutation load than MSS tumors in this CRC cohort (*p* < *0.0001*, Fig. 2a). These patients (MSI-H) also had higher cytolytic activity (as denoted by CYT) than those with MSS tumors, as shown by the jitter plot (Fig. 2b). Thus, indicating high cell killing activity intra-tumorally, most likely due to immune cell infiltration. We also measured intra-tumoral genetic heterogeneity by determining the MATH score for MSI-H compared to MSS patients. MSI-H tumors demonstrated significantly lower MATH than MSS (*p* < *0.0001*, Fig. 2c).

### MSI-H tumors possess higher composition of tumor infiltrating immune cells

**Figure 3:**
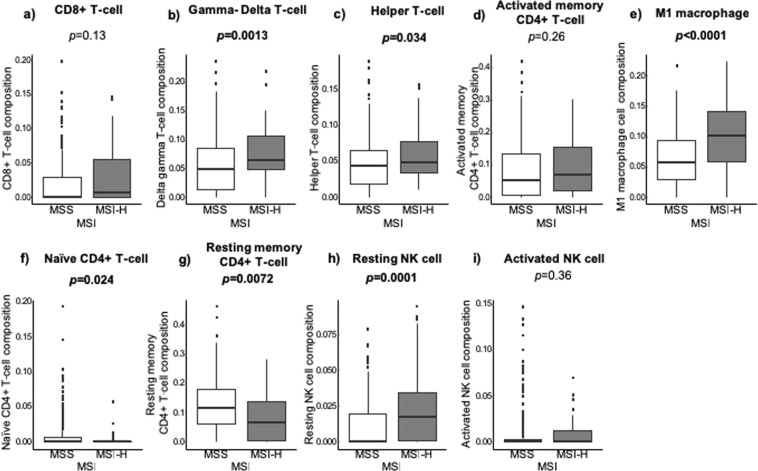
MSI-H vs. MSS in (**a**) CD8+ T-cell, (**b**) Gamma-Delta T-cell, (**c**) Helper T-cell, (**d**) Activated memory CD4+ T-cell, (**e**) M1 macrophage, (**f**) Naïve CD4+ T-cell, (**g**) Resting memory CD4+ T-cell, (**h**) Resting NK cell and (**i)** Activated NK cell.

Given the high cytolytic activity score in MSI-H tumors, it was of interest to examine which immune cells are infiltrated in MSI-H patients compared to MSS utilizing the CIBERSORT algorithm (Fig. 3). T-cell expression was significantly higher in the MSI-H group compared to MSS in Gamma-Delta T-cell (*p* = *0.0013*) and Helper T-cell groups (*p* = *0.034*). This trend was also identified (without achieving statistical significance) in the CD8+ T-cell (*p = 0.13*) and Activated Memory CD4+ T-cell (*p* = *0.26*) groups. MSS was associated with higher expression of Naïve CD4+ T-cells (*p* = *0.024*) and Resting memory CD4+ T-cells (*p* = *0.0072*). There was no difference between the two groups when measuring T regulatory (T-reg) cells (*p* = *0.96*). MSI-H patients also had a greater fraction of M1 type macrophages than MSS (*p* < *0.0001*) as well as more resting NK cells (*p* = *0.0001*). There, however, was no difference between MSI-H and MSS groups when measuring for activated NK cells (*p* = *0.36*).

### MSI-H associates with high expression of immune-response related genes and immune checkpoint molecules (ICM)

**Figure 4:**
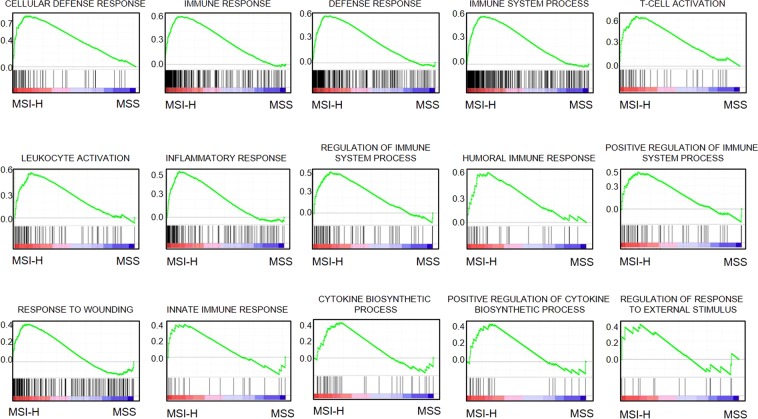
Gene Sets Enrichment Analysis (GSEA) inclusion of 15 available immune-response related gene sets which were significantly upregulated in the MSI-H CRC tumors and diminished in MSS.

Gene Sets Enrichment Analysis (GSEA) was conducted to validate the association between CYT and immune-response signatures in MSI-H and MSS tumors (Supplementary Table 1). GSEA using TCGA dataset identified 15 available immune-response related gene sets, which were significantly upregulated in the MSI-H CRC tumors; suggesting that MSI-H positively associated with intra-tumoral immune response in MSI-H tumors (Fig. 4).

**Figure 5:**
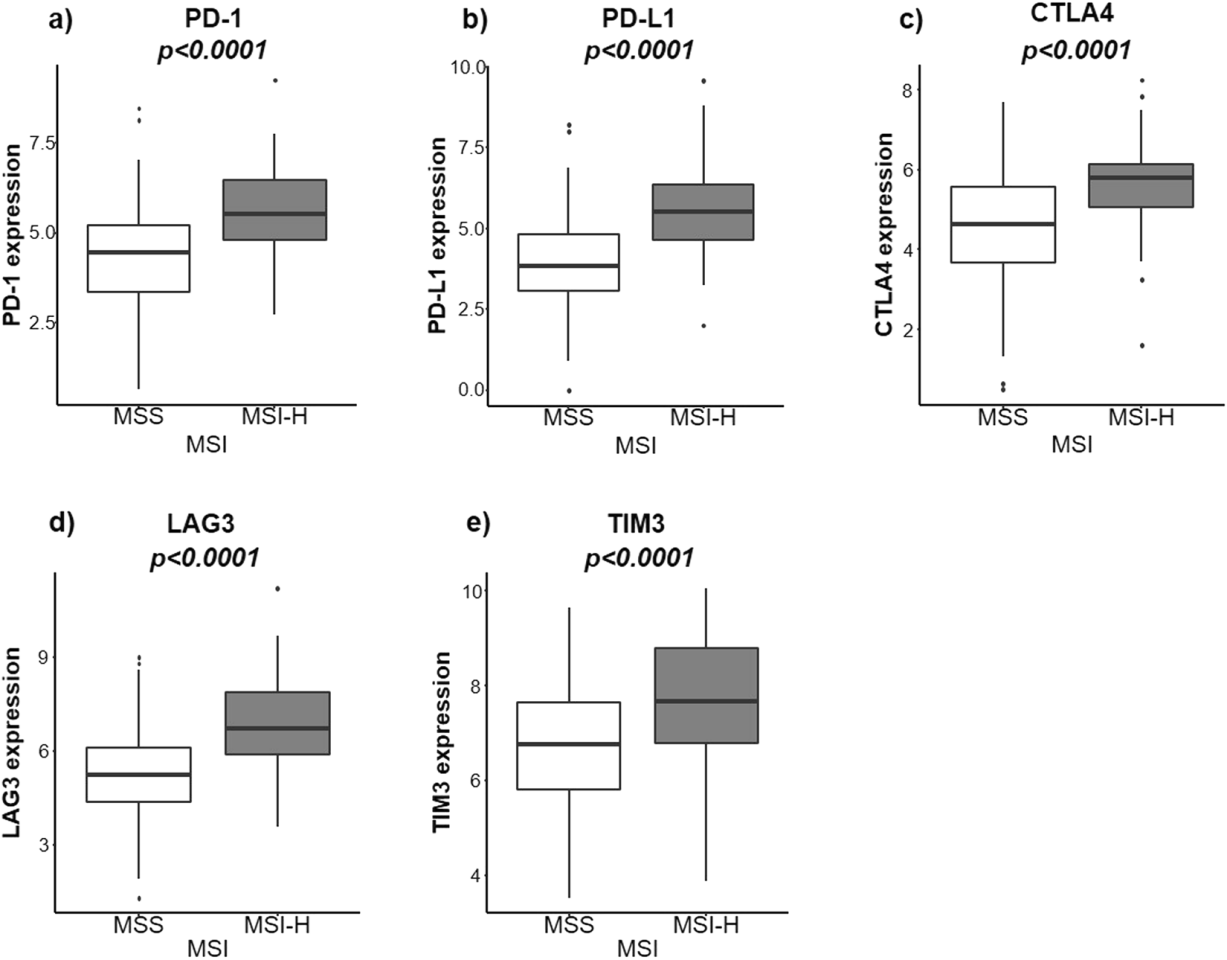
MSI-H vs. MSS and their association with immune checkpoint molecules: (**a**) PD-1, (**b**) PD-L1, (**c**) CTLA4, (**d**) LAG3 and (**e**) TIM3.

MSI-H and MSS tumors were then compared for expression of immune checkpoint molecules (ICM) including PD-1, PD-L1, CTLA4, LAG3 and TIM3 (Fig. 5). The expression (relative values) of all of these molecules was higher in in the MSI-H group than MSS: PD-1 (5.53 vs. 4.44, *p* < *0.0001*), PD-L1 (5.52 vs. 3.83, *p* < *0.0001)*, CTLA4 (5.80 vs. 4.62, *p* < *0.0001*), LAG3 (6.71 vs. 5.24, *p* < *0.0001*) and TIM3 (7.66 vs. 6.76, *p* < *0.0001*).

### Impact of MSI-H and DNA repair gene expression on survival

**Figure 6:**
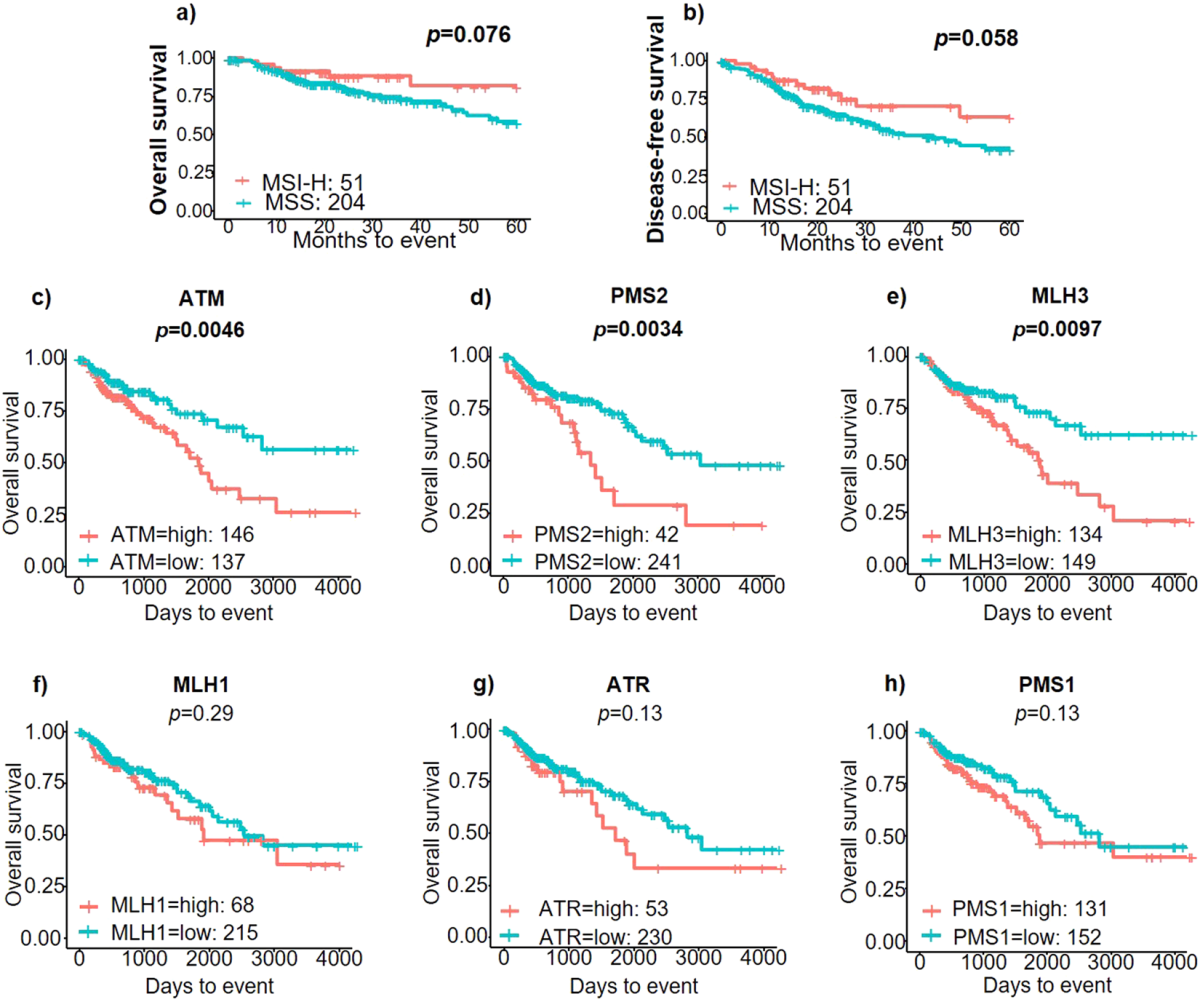
Survival analysis of MSI-H vs. MSS in CRC patients: (**a**) Kaplan-Meier (KM) Curve of Overall Survival (OS) with MSI-H vs. MSS, (**b**) KM Curve of Disease-Free Survival (DFS) with MSI-H vs. MSS, KM Curves of Overall Survival (OS) comparing high vs. low expression of DNA repair genes: (**c**) ATM, (**d**) PMS2, (**e**) MLH3, (**f**) MLH1, (**g**) ATR and (**h**) PMS1.

We then studied whether MSI status, or DNA repair gene expression associated with patient survival. MSI-H patients trended towards having higher 5-year OS (79.4% vs. 59.5%, *p* = *0.076*) and DFS (59.7% vs. 43.6%, *p* = *0.058*) than the MSS cohort without achieving statistical significance (Fig. 6a,b). In further survival analysis, broken down by stage we did note that MSI-H patients had trended towards improved 5-year OS than MSS in all stages. This was most markedly pronounced in stage II (92% vs. 72.6%, *p* = *0.5*) and stage III (66.7% vs. 47.6%, *p* = *0.24*). There were also trends towards improved DFS in MSI-H compared to MSS in stage I (83.3% vs. 62.3%, *p* = *0.77*), stage III (60% vs. 38.9%, *p* = *0.26*) and stage IV (50% vs. 10.1%, *p* = *0.9*). These did not achieve statistical significance likely secondary to smaller patient numbers. We also found significantly improved 5-year OS in patients with low expression of certain DNA repair genes compared to high expression of these genes, which included *ATM* (74.4% vs. 51.9%, *p* =  *0.004*), *PMS2* (72.6% vs. 28.8%, *p* =  *0.003*) and *MLH3* (73.7% vs. 52.3%, *p* = *0.0097*), (Fig. 6c,e). There was also a trend towards improved 5-year survival in patients with low expression of *MLH1* (66.3% vs. 57.1%, *p* =  *0.29*), *ATR* (68.3% vs. 46.7%, *p = 0.13*) and *PMS1* (71.3% vs. 54.6%, *p* =  *0.13*), (Fig. 6f–h).

### Impact of MSI and immune checkpoint molecule expression on survival

**Figure 7:**
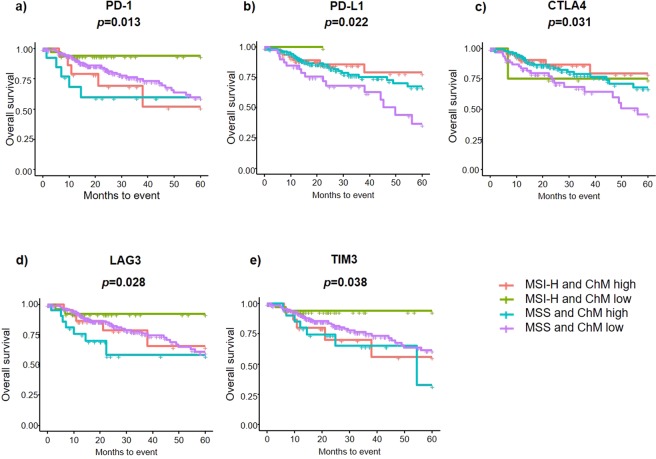
Survival analysis of MSI-H vs. MSS in CRC patients stratified by their association with immune checkpoint molecules: (**a**) PD-1, (**b**) PD-L1, (**c)** CTLA4, (**d**) LAG3 and (**e**) TIM3. High, high expression of each gene; low, low expression of each gene.

We then investigated the survival of patients in MSI-H and MSS groups, measuring for concurrent expression of ICMs which are known to function as “brakes” of immune response. We found that MSI-H patients with low ICM expression had significantly improved 5-year OS compared to MSS patients with high ICM expression (Fig. 7). This association was identified in ICMs: PD1 (93.9% vs. 59.8%, *p* = *0.022*), CTLA4 (75% vs. 67.6%, *p* = *0.031*), LAG3 (92.3% vs. 58.1%, *p* = *0.028*) and TIM3 (93.9% vs. 32.5%, *p* = *0.038*).

## Discussion

In this study, we used a novel completely bioinformatic approach using unbiased RNA-sequencing data from the TCGA CRC data set to perform in-depth analyses of the tumor immune microenvironment of MSI-H vs. MSS patients and to confirm findings of increased intra-tumoral immunogenicity in MSI-H patients associating with improvement in clinical outcomes. We, using this unique methodology, were able to reach concordant results with studies which utilized conventional methods of evaluating tumor immunology such as immunohistochemistry or flow cytometry but with diminished costs, lower time and labor expenditure and with higher reproducibility.

Secondary to the diminished DNA repair mechanisms in MSI-H CRC, somatic mutations accumulate within coding and non-coding regions in DNA^4^. As the reading frames of oncogenes or tumor suppressors are altered, tumors are generated^4^. This impaired DNA repair and genomic instability can lead to increased neoantigen load on the surface of tumor cells which make them more prone to attack by lymphocytes and other immune cells than MSS tumors^30^. In our evaluation of TCGA CRC cohort, we found that MSI-H patients trended towards having improved OS and DFS compared with the MSS cohort, a finding which is consistent with previously published data^31–33^. This trend appeared to be most pronounced in examining OS in stage II and stage III patients and less so in stage IV (likely due to low patient numbers in this group). We also found that MSI-H patients had a higher mutation load as well as high CYT compared to MSS, likely secondary to prominent lymphocytic infiltrate resulting in elevation in intra-tumoral immune cytolytic activity similar to what has been observed previously in different settings^12,22,30,34^.

In gene expression analysis from TCGA we identified several DNA repair genes and determined that low expression of MMR deficient genes including *MLH1*, *MLH3*, *PMS1* and *PMS2* as well as other double stranded break DNA repair genes including *ATR, PRKDC*, *ATM* and *BRCA2* was associated with MSI-H. We also found improved 5-year survival in patients with lower expression of several of these genes including *ATM, PMS2, MLH3, PMS1, MLH1* and *ATR* in comparison to survival in patients with MSI-H tumors which did not achieve statistical significance, likely due to fewer numbers of patients and a too short follow up. The fact that expression of DNA repair genes reached statistical significance may indicate that they may be stronger prognostic biomarkers.

For MSI-H CRC as well as other immunogenic cancers, a high level of T lymphocyte infiltration into tumors has been noted to be a positive prognostic factor^11^. MSI-H tumors are infiltrated with intra-epithelial cytotoxic T-cells and activated CD4+ helper T-cells, making them increasingly prone to a local cytotoxic immune response^35^. We noted this same association in the patients of this study with MSI-H tumors being significantly associated with infiltration by helper T-cells as well as trending towards increased infiltration by cytotoxic (especially Gamma-Delta) and activated memory CD4+ T-cells. There was, in our study, no difference between MSI-H and MSS groups in the expression of T-reg lymphocytes. Other studies have noted that increased expression of T-reg cells compared to CD4+ and CD8+ lymphocytes can indicate a poorer outcome likely due to suppression of cytotoxic T-cells^35,36^.

We also found in this study that MSI-H patients had a higher ratio of intra-tumoral M1 macrophages than the MSS group. M1 macrophages have been demonstrated previously to be associated with the inflammatory response via release of pro-inflammatory cytokines as well as pathogen clearance and anti-tumor immunity^37^. M1 macrophages have also been shown in previous studies to have tumor suppressive effects via production of reactive oxygen species which we hypothesize also may have contributed to the trend in improved survival in MSI-H patients^38^.

MSI-H tumors were also found to have elevated tumor mutation burden but diminished intra-tumoral heterogeneity as defined by MATH than MSS. This may have contributed to improvement in survival in MSI-H patients^26^. There has been increasing interest in increasing genetic diversity within tumors resulting in clonal evolution as a response to anti-tumor immunosurveillance^39–41^. We may speculate that MSI-H patients have low tumor heterogeneity due to increased clonal selective pressures from robust immunologic responses within these tumors.

Immune checkpoints are an immune inhibitory mechanism by which cancer cells evade anti-tumor immunity^42,43^. Some immune checkpoint molecules have been identified as potential targets for immunotherapy. These include PD-1 (programmed cell death molecule), PD-L1 (PD1 ligand), CTLA-4 (cytotoxic T-lymphocyte associated protein 4), LAG-3 (lymphocyte activation gene) and TIM3, an inhibitory molecule selectively expressed on IFN-γ-producing helper and cytotoxic T-cell responses^44–47^. This study found that expression all of these molecules (PD-1, PD-L1, CTLA4, LAG3 and TIM3) was higher in in the MSI-H group than MSS which may be a result of the immune activation driven by effector T-cells in patients with MSI-H tumors.

PD-1/PD-L1 binding has been demonstrated to block the effector function and motility of most lymphocytes, thereby decreasing the production of IL-2 (interleukin-2) by helper T-cells and diminishing the clonal proliferation of cytotoxic T-cells in response to cancer cells^44^. This impaired immune function is termed “T-cell exhaustion” and allows cancer cells to escape immune surveillance^11,44^. Studies have also noted that high PD-1 and PD-L1 expression on tumor cells has been associated with a weakened host immune response and subsequent poor prognosis in a number of malignancies^42,45,48^. Up-regulation of PD-L1 has been reported in several malignancies including CRC, melanoma, lung cancer, renal cell carcinoma, ovarian cancer, breast cancer and osteosarcoma^48^. It has been associated with more frequent incidence of vascular invasion, tumor recurrence and lower numbers of cytotoxic T-cells^48^. We identified a similar trend within this study, finding that MSS patients with elevated ICM expression had significantly poorer survival than MSI-H patients with lower ICM expression.

Recently, treatment with immune checkpoint inhibitors such as anti-PD1 antibodies (e.g. Nivolumab and Pembrolizumab) and its ligand anti-PD-L1 (e.g. Atezolizumab) have been increasingly used as an effective treatment strategy to combat various advanced cancers^11,49^. Le *et al*. in their phase II trial examining anti-PD-1 blockade found a significantly improved objective response rate and survival in MSI-H CRC compared to MSS with associated TIL elevation^49^. This is likely a result of MSI-H CRCs association with increased neoantigen (immunogenic tumor mutated peptide) expression, concurrent with PD-1 inhibition; resulting in elevated TIL expression and tumor regression^35,45^. Targeting MSS tumors in advanced CRC may present a more difficult challenge with decreased responsiveness to immunotherapy and would thus be more likely to be treated with standard chemotherapy regimens or via methodologies being developed to increase intra-tumoral immunogenicity (such as PARP inhibitors or vaccines) in combination with immunotherapeutic agents^50–52^.

Some limitations of TCGA data included limited clinical information regarding patients’ co-morbid conditions and therapeutic information. We were also only able to make associations between these analyses of RNA sequencing data and clinical outcomes from TCGA without being able to elucidate underlying molecular mechanisms or make direct correlations. Also, the majority of patients had locoregional disease which likely contributed to the improved survival of these patients. Additionally, the TCGA was created prior to the wide use of immune checkpoint inhibition, thus our data reflects patients who did not receive those treatments.

## Conclusions

This study used a unique bioinformatic approach to analyze RNA sequencing data, obtained from The Cancer Genome Atlas to identify disparities within the tumor immune microenvironments of MSI-H and MSS colorectal cancer patients. Using this approach, we were able to find an association between low expression of non-mismatch DNA repair genes as well as known MMR genes with high microsatellite instability. We found that MSI-H was associated with high TIL and M1 macrophage infiltration into the tumor immune microenvironment as well as with higher cytolytic activity and diminished heterogeneity (with likely increased clonality) which may have contributed to the improvement in survival. We also found an association between MSI-H and 15 different immune response gene signatures as well as immune checkpoint molecules which can be used to further development of targeted therapies (not only in ICMs which already have immunotherapies such as PD-1, PD-L1 and CTLA4, but also in future therapies targeting LAG3 and TIM3 which are currently targeted by no immunotherapeutic agents). The primary novelty of our study is that it allowed us to utilize a completely bioinformatic approach to perform an in-depth analysis of the tumor immune microenvironment using RNA sequencing data with low associated costs, increased feasibility and increased reproducibility than conventional methods of studying tumor immunology.

## Supplementary information

Supplementary Figures

## Data Availability

There are no restrictions on the availability of materials or data for this project. Data was obtained from the publicly available The Cancer Genome Atlas.
