# Supplementary material for: Tumor Infiltrating Lymphocytes and Macrophages Improve Survival in Microsatellite Unstable Colorectal Cancer
Source: Sci Rep. 2019 Sep 17;9:13455. doi: 10.1038/s41598-019-49878-4 (PMC6748965; doi:10.1038/s41598-019-49878-4)

**Tumor Infiltrating Lymphocytes and Macrophages Improve Survival in Microsatellite Unstable Colorectal Cancer**

Sumana Narayanan**^1^, MD., Tsutomu Kawaguchi**^1^, MD, Ph.D., Xuan Peng^2^, MS., Qianya Qi^2^, MS., Song Liu^2^, PhD., Li Yan^2^, PhD., and Kazuaki Takabe*^1,3^, MD, Ph.D, FACS

** These authors contributed equally to this work.

^1^ Department of Surgical Oncology, Roswell Park Cancer Institute, Buffalo, NY, USA

^2^ Department of Biostatistics & Bioinformatics, Roswell Park Cancer Institute, Buffalo, NY, USA

^3^ Department of Surgery, Jacobs School of Medicine and Biomedical Sciences, University at Buffalo, The State University of New York, Buffalo, NY, USA

**Supplementary Figure S1-** Kaplan-Meier (KM) Curves of Overall Survival (OS) comparing high vs. low expression of DNA repair genes: a) ATM, b) PMS2, c) MLH3, d) MLH1, e) ATR and f) PMS1.


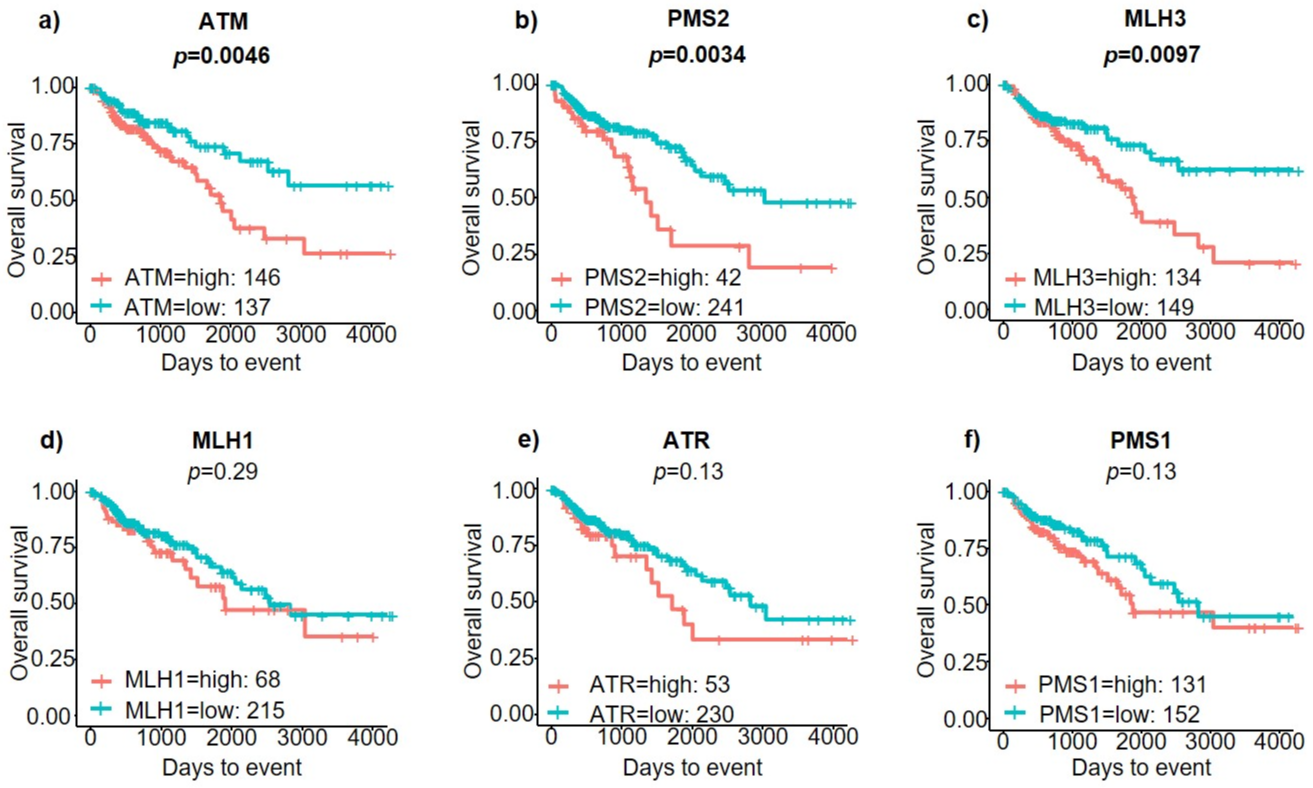


**Supplementary Table 1-** Gene Sets Enrichment Analysis (GSEA) including 15 available immune-response related gene sets, which were significantly upregulated in the MSI-H CRC tumors. ES- enrichment score; NES- normalized enrichment score; NOM p-value- normalized p-value; FDR- false discovery rate.


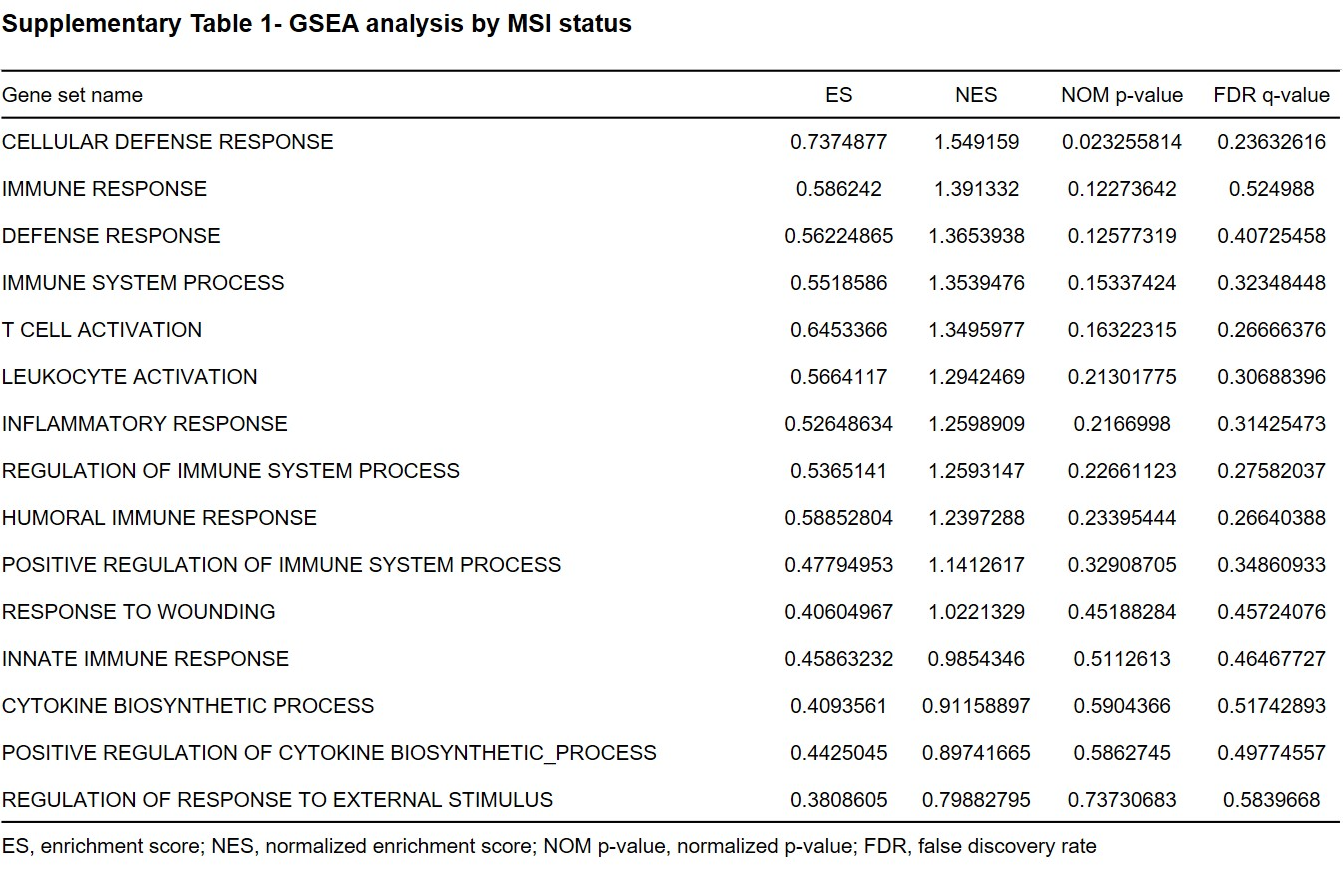

Supplement: Supplementary file 1 — Supplementary Figures [file 41598_2019_49878_MOESM1_ESM.docx]
